# Evaluation of Features in Probably Benign and Malignant Nonmass Enhancement in Breast MRI

**DOI:** 10.1155/2024/6661849

**Published:** 2024-03-15

**Authors:** Nasrin Ahmadinejad, Fahimeh Azizinik, Pershang Khosravi, Ala Torabi, Amirhassan Mohajeri, Arvin Arian

**Affiliations:** ^1^Advanced Diagnostic and Interventional Radiology Research Center (ADIR), Imam Khomeini Hospital, Cancer Institute, Tehran University of Medical Sciences, Tehran, Iran; ^2^Advanced Diagnostic and Interventional Radiology Research Center (ADIR), Imam Khomeini and Yas Hospital, Cancer Institute, Tehran University of Medical Sciences, Tehran, Iran; ^3^Department of Radiology, Imam Khomeini Hospital, Tehran University of Medical Sciences, Tehran, Iran; ^4^Department of Radiology, Shariati Hospital, Tehran University of Medical Sciences, Tehran, Iran; ^5^Islamic Azad University of Medical Science, Tehran, Iran

## Abstract

Dynamic contrast-enhanced magnetic resonance imaging (DCE-MRI) is a highly sensitive breast imaging modality in detecting breast carcinoma. Nonmass enhancement (NME) is uniquely seen on MRI of the breast. The correlation between NME features and pathologic results has not been extensively explored. Our goal was to evaluate the characteristics of probably benign and suspicious NME lesions in MRI and determine which features are more associated with malignancy. We performed a retrospective research after approval by the hospital ethics committee on women who underwent breast MRI from March 2017 to March 2020 and identified 63 lesions of all 400 NME that were categorized as probably benign or suspicious according to the BI-RADS classification (version 2013). MRI features of NME findings including the location, size, distribution and enhancement pattern, kinetic curve, diffusion restriction, and also pathology result or 6-12-month follow-up MRI were evaluated and analyzed in each group (probably benign or suspicious NME). Vacuum-guided biopsies (VAB) were performed under mammographic or sonographic guidance and confirmed with MRI by visualization of the inserted clips. Segmental distribution and clustered ring internal enhancement were significantly associated with malignancy (*p* value<0.05), while linear distribution or homogeneous enhancement patterns were associated with benignity (*p* value <0.05). Additionally, the plateau and washout types in the dynamic curve were only seen in malignant lesions (*p* value <0.05). The presence of DWI restriction in NME lesions was also found to be a statistically important factor. Understanding the imaging findings of malignant NME is helpful to determine when biopsy is indicated. The correlation between NME features and pathologic results is critical in making appropriate management.

## 1. Introduction

Dynamic contrast-enhanced magnetic resonance imaging (DCE-MRI) is a highly sensitive breast imaging modality with approximately 90% or higher sensitivity in detecting breast cancer. However, MRI has lower specificity [1–4].

The indication for breast MRI has greatly increased, and it has become an important tool for diagnosing and evaluating breast cancer. Currently, the more common clinical indications include screening in high-risk women and assessment of indeterminate breast lesions that were detected with other imaging modalities, such as mammography or ultrasound, response evaluation to neoadjuvant chemotherapy, and preoperative staging in patients with known breast cancer [5–9]. The BI-RADS lexicon is the main reference to breast imaging terminology, reporting standards, and classification systems for mammography, ultrasound, and MRI of the breast. Lesions detected by MRI can be classified by morphology into mass, nonmass enhancement, or focus. If the enhancement is neither focus nor mass, it is determined as nonmass enhancement (NME). NME is defined as an area of enhancement that is distinct from the surrounding background parenchymal enhancement, without any corresponding space-occupying mass in precontrast images including T1, T2, or DWI images [10]. According to the latest BI-RADS lexicon, the distribution pattern of NME includes focal, linear, segmental, regional, multiple regions, and diffuse. Internal enhancement patterns in NME are classified as homogeneous, heterogeneous, clumped, and clustered ring. A wide spectrum of benign and malignant pathologies has been reported in NME cases [11–15]. The correlation between NME features and pathologic results has not been extensively evaluated, and due to overlapping between benign and malignant lesions, concordance between MRI and pathologic findings in such cases may be unclear, which could result in the need for additional workup [16]. The main goal of our study was to evaluate the characteristics of imaging findings in probably benign and suspicious NME on MRI and to determine which features are more associated with malignancy.

Understanding the characteristics in breast cancer prediction for each NME descriptor will improve the detection rate.

## 2. Materials and Methods

This study was performed after approval by the hospital ethics committee. Specific written consent was waived due to the retrospective nature of the study.

All patients who undergone breast MRI from March 2017 to March 2020 in our academic tertiary breast center were identified from the Picture Archiving and Communications System (PACS). The inclusion criteria were NME findings that were labeled as BI-RADS 3 (probably benign) or BI-RADS 4 and 5 (suspicious) categories, having short-term (6 months and then 12 months) follow-up dynamic breast MRI or pathological diagnosis, respectively. Patients without complete medical or imaging files were excluded. Patients with benign pathology report were followed with dynamic MRI after 6 months.

Finally, 63 patients who had probably benign or suspicious NME in MRI were included in our study. Two radiologists with breast imaging fellowship degree and at least 10 years of experience independently evaluated the imaging features of NME in breast MRI imaging, and they were blinded to the pathology reports. In the case of discordance, a more experienced breast radiologist made the final decision.

Patients' data including age, screening or diagnostic setting, risk factor or family history of breast cancer, biopsy results, and MRI characteristics of NME findings including the location, size, distribution, and internal enhancement pattern of NME lesions, dynamic curve type, presence/absence of diffusion restriction, and ADC value number were evaluated in both groups.

Lesion location was determined as UOQ, UIQ, LIQ, LOQ, and central or retroareolar regions.

According to the latest BI-RADS lexicon, the distribution pattern of NME was classified as focal, linear, segmental, regional, multiple regions, and diffuse.

Internal enhancement patterns in NME were categorized as homogeneous, heterogeneous, clumped, and clustered ring.

Time-intensity curve (TIC) was assessed in two parts: the initial and delayed phase.

The initial phase is described as an increase of signal intensity within 2 minutes to three categories: slow (less than 50%), medium (50-100%), and fast categories (more than 100%). After 2 minutes, the delayed phase is described as persistent (an enhancement increase more of than 10% over time), plateau (stable enhancement or less than 10% change over time), and washout (more than 10% signal reduction overtime).

Based on the abovementioned characteristics, NME cases were classified as one of the following three types of enhancement curves: type 1 (progressive enhancement pattern); type 2 (plateau pattern), and type 3 (washout pattern) [10]. ADC value number was calculated with ROI (region of interest) in the lowest signal area of nonmass enhancement excluding hemorrhage or cystic regions.

Imaging parameters were as follows: for axial T1 images without contrast, repetition time/echotimeTR/TE467/3, FA: 10°, FOV: 320-350 mm, matrix: 320 × 320, NEX: 1, and ST: 2.6 mm; for axial T2 images, (TR/TE) = 5000 − 6000/102 − 105, FOV: 320-350 mm, matrix: 384 × 256, NEX: 2, and ST: 5 mm; for dynamic axial gradient fat-suppressed T1 images, TR/TE = 5 − 6/1.5 − 2, FA: 10°, FOV: 320-350 mm, matrix: 350 × 350, NEX: 1, and ST: 2 mm; and for echo-planar imaging- (EPI-) based diffusion-weighted imaging (DWI), TR/TE3000/65, FOV: 320-350 mm, matrix: 192 × 192, NEX: 4 and ST: 4 mm, and *b*-value = 800.

Probably benign (BI-RADS 3) and suspicious (BI-RADS 4 or 5) NME cases on MRI were classified as in Table 1.

Biopsy was performed for lesions with any suspicious feature in MRI including linear or segmental distribution of enhancement, clumped or clustered ring pattern of enhancement, restricted diffusion, and finally, kinetic curves with fast early enhancement and delayed plateau or washout pattern.

Although MRI-guided biopsy was optimal for NME lesions, vacuum-assisted biopsy under ultrasound or mammography guidance was performed, and subsequently, proper localization was confirmed by postbiopsy nonenhanced fat-suppressed T1-weighted images. Short-term follow-up was performed for the NMEs with probably benign features in 6-12 months as focal or regional distribution of enhancement, heterogeneous or homogenous enhancement pattern, or medium/slow early enhancement with persistent delayed curve [17].

In our study, one of the NME lesions lacks the corresponding finding in the ultrasound study or mammogram which was followed up in 6 and 12 months without significant change.

According to changes in the follow-up period, the lesions were grouped as stable, regression, or progression. The lesions which were stable or showed regression during this period were considered as benign (BI-RADS 2).

To determine differences in MRI features between benign and malignant NME cases, Pearson's *χ*2 test, Fisher's exact test, and independent *t* test were used. For comparison of qualitative variables, the chi-square test was used.

A *p* value <0.05 was considered statistically significant. Statistical analysis was performed by using SPSS (version 23).

## 3. Results

Of all 400 NME lesions in MRI, 337 patients were excluded because of history of previous treatment, incomplete medical data, and poor-quality images. Finally, we enrolled 63 cases with probably benign or suspicious NME into the study according to the BI-RADS classification (version 2013). Of 63 NME cases, 18 (28.5%) malignant lesions were detected including 11 (61.2%) invasive ductal carcinoma, 4 (22.3%) DCIS, 1 (5.5%) LCIS, 1 (5.5%) invasive lobular carcinoma, and 1 (5.5%) inflammatory carcinoma.

The majority of NME lesions were benign in pathology including 45 cases (71.5%).

The patients ranged from 20-69 years of age with a mean age of 41.7 years (±1.1). The mean age for patients with malignant pathology results was 46.9 years and for patients with benign pathologies was 40.9 years which shows statistically significant difference (*p* value = 0/036).

The most common pathologies in benign NME lesions were fibrocystic change spectrum, sclerosingadenosis, intraductal papilloma, and inflammation.

Of all 63 NME cases, 9 (14.3%) had a positive family history of breast cancer, 8 (12.7%) had a positive personal history of breast cancer, and 46 (73%) had no family history. Only 3 (16.6%) malignant NME cases were found in patients with a personal history of breast cancer, so the majority of the malignant lesions were in patients without personal or family history of breast cancer.

The mean size of NME lesions in malignant cases was 48.2 mm and in benign results was 41.2 mm which was not statistically significant between the two groups (*p* value = 0.613).

In respect with NME location (UOQ, UIQ, LIQ, LOQ, and central or retroareolar regions) in malignant and benign NME cases, no statistically significant was seen.

In the overall study population group, 24 (38%) of lesions had been classified as BI-RADS 3, 35(56%) as BI-RADS 4, and 4 (6%) as BI-RADS 5. One case of BI-RADS 3 proved malignant in follow-up to be DCIS.

Of the 24 NME categorized as BI-RADS 3, 23 lesions were benign in pathology or follow-up yielding ppv of 4% for malignancy. Of all NME labeled as BI-RADS 4, 63% were benign, and 37% were malignant in pathology with ppv of 37%. All NME labeled as BI-RADS 5 were malignant in pathology results yielding a ppv of 100%.

The most frequent distribution pattern was focal (28; 44.5%), followed by segmental (20; 31.8%), linear (11; 17.5%), regional (2; 3.1%), and diffuse (2; 3.1%) patterns.

Among the distribution patterns of the NME lesions, segmental distribution, linear, and focal distribution were found to be statistically different between the benign and malignant lesions (*p* value <0.05). The segmental distribution in malignant NME lesions (66.7%) was more common than that in the benign lesions (17.7%) (*p* value <0.0001), and all linear distribution patterns in NMEs were benign in pathology results (*p* value = 0.01).

The focal distribution pattern was seen in 53.3% of benign NMEs, while 22.2% of malignant NMEs showed focal distribution, and this was statistically different (*p* value = 0.023). Of 13 NME lesions with pathology-proven invasive carcinoma, 7.7% showed the focal distribution pattern, and in contrast, 60% of NMEs with carcinoma in situ depicted focal distribution (*p* value = 0.044).

Regarding the internal enhancement feature in NMEs, the most frequent internal enhancement pattern was heterogeneous (49.4%), followed by homogenous (30.1%), clumped (14.2%), and clustered ring (6.3%).

**Figure 1:**
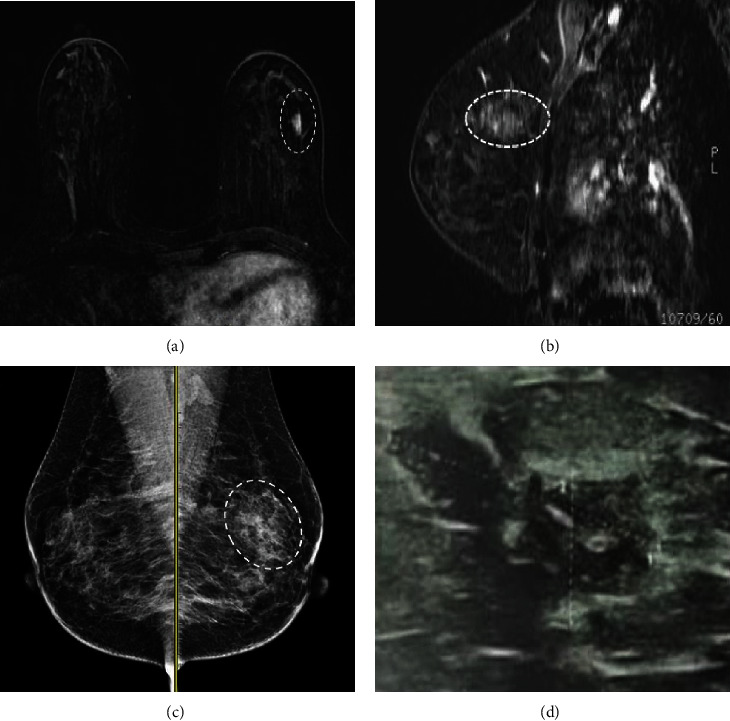
(a, b) Axial- and sagittal-subtracted contrast-enhanced breast MRI shows focal heterogenous NME in the upper outer aspect of the left breast (dashed circle). In MLO views (c), a focal asymmetry is seen (CC views are not shown) without microcalcification or architectural distortion. In targeted ultrasound (d), focal heterogenous fibroglandular tissue was identified. Pathology proved benign fibrocystic changes.

The frequency of internal enhancement pattern in malignant NMEs was heterogeneous (55.5%), followed by clustered ring (22.3%), clumped (16.7%), and homogenous (5.5%) (Figure 1). The frequency of internal enhancement pattern in benign NMEs was heterogeneous (46.7%), followed by homogenous (40%), and clumped (13.3%).

**Figure 2:**
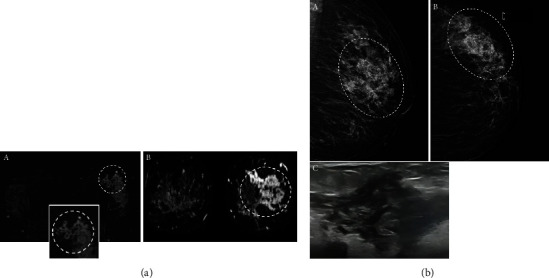
(a) Axial- and coronal-subtracted contrast-enhanced breast (A, B) MRI images in a 46-year-old female show clustered ring nonmass (dashed circle) enhancement with segmental distribution in the left breast. The cluster ring internal enhancement pattern is better visible in the inset photo. The biopsy result was invasive ductal carcinoma. (b) (A) MLO and (B) CC views of the same patient show segmental pleomorphic microcalcifications (dashed circle). (C) Targeted ultrasound demonstrates a nonmass lesion with tubular structures and architectural distortion.

All NME lesions with clustered ring internal enhancement pattern were malignant; in other words, none of the 45 benign NME cases shows clustered ring enhancement, and this difference was statistically significant (*p* value = 0.001) (Figure 2).

Homogenous enhancement pattern was seen in 40% of benign NMEs, while the majority of the malignant NME (94.4%, 17 of 18) did not show a homogenous pattern, and this difference was statistically significant (*p* value = 0.007). Other types of internal enhancement were not statistically significant in malignant and benign groups.

With respect to the enhancement dynamic curve, of all 63 NME cases, 48 lesions showed type 1 enhancement kinetic curve (76%), 10 lesions had type 2 (16%), and 5 lesions showed type 3 (8%).

All 45 benign NMEs had type 1 kinetic curve, and all type 2 and 3 kinetic curves were malignant in pathology results with statistically significant difference. (*p* value <0.0001).

The type of dynamic curves between invasive cancer and carcinoma in situ was also evaluated. None of the noninvasive NMEs demonstrated washout dynamic type, while some of the invasive carcinoma (38.5%) had type 3 dynamic curve; however, it was not statistically significant (*p* value = 0.1).

**Figure 3:**
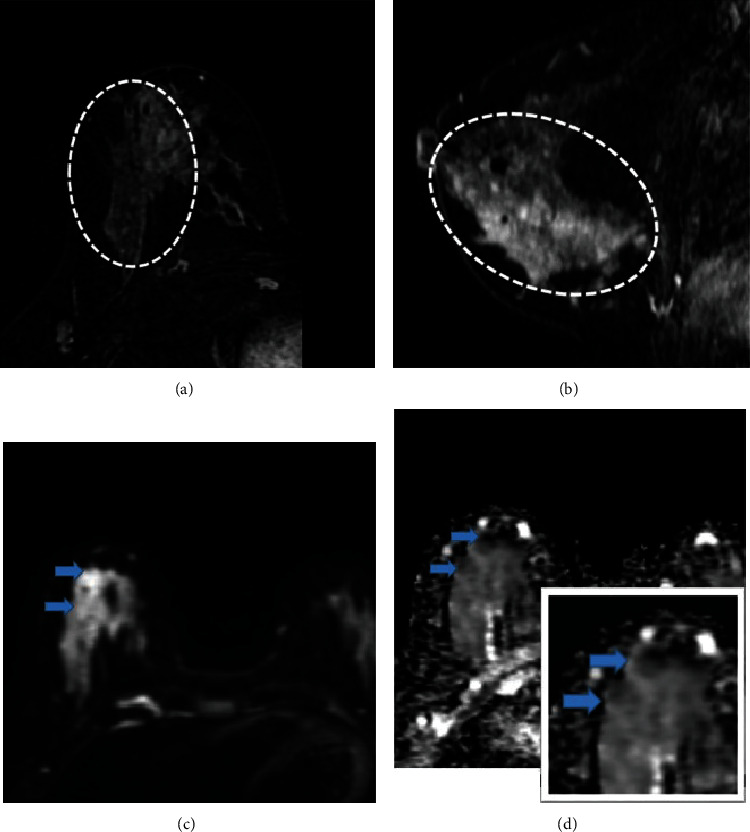
(a, b) Axial and sagittal contrast-enhanced breast MRI images in a 47-year-old female with pathology-proven invasive ductal carcinoma and ductal carcinoma in situ, depicting segmentally distributed clumped nonmass enhancement (dashed circle) in the right breast. (c, d) DWI and ADC images show heterogenous restriction which is better demonstrated in the inset image.

The presence of diffusion restriction on DWI sequences was seen in 89% of malignant NMEs (Figure 3). Only 4.4% of benign NME lesions showed diffusion restriction which was statistically significant (*p* value <0.0001).

The mean ADC value number in malignant NME lesions was 1.28 ± 0.14 × 10 − 3 mm^2^/s and in benign NMEs was 1.63 ± 0.16 × 10 − 3 mm^2^/s which was also statistically significant (*p* value <0.0001).

Restricted diffusion on DWI was seen in all invasive carcinoma and in 40% noninvasive cancer (*p* value = 0.06).

Additionally, the average of ADC values in invasive cancer (1.25 × 10 − 3 mm^2^/s) was lower than that in carcinoma in situ (1.36 × 10 − 3 mm^2^/s), but their differences were not statistically significant (*p* value = 0.17).

MR imaging descriptors in NME lesions are demonstrated in Table 2.

## 4. Discussion

DCE-MRI is a highly sensitive breast imaging method for detecting breast cancer and is used for various indications [16].

The correlation between NME characteristics and pathological findings has not been comprehensively evaluated, and as benign and malignant lesions overlap, the agreement between MRI and pathological findings may be unclear in such cases and require additional investigation.

Therefore, the purpose of this study was to evaluate different imaging features of benign or malignant lesions that appear as NMEs on MRI and to determine which features can more reliably distinguish malignant from benign lesions.

Understanding each NME descriptor may help predict malignant or benign NME and improve breast cancer detection rate.

In our study, most NME lesions, 45 of 63 (71.5%), were found to be pathologically benign, and 18 of 63 (28.5%) of NME cases were found to be malignant. These results are similar to several recent studies.

In the study by Torous et al., 61.5% of all NME cases were benign, and 22.3% (29 of 130) of cases corresponded to DCIS or invasive carcinoma [16]. Jabbar et al. also found that a minority of NME cases were malignant on pathological examination (14%, 11 of 76 cases) [14].

In contrast, in the study by Jansen et al., the majority of NME lesions were associated with malignant lesions (81.2%, 212 of 261 cases) [18].

The study by Ballesio et al. also showed that the majority of NME cases represented malignant lesions (73.4%, 69 of 94 lesions) [13].

Therefore, our study shows that a wide variety of lesions, from benign to malignant, can be depicted as NMEs on MRI.

Morphological features, including internal enhancement and distribution patterns, are the most important features for the description of NMEs [19].

In respect to distribution pattern, Asada et al. [20] found that segmental distribution was significantly associated with malignancy (*p* value <0.05), which was similar to the results of our study, in which the majority of malignant NME cases (66.7%) presented as segmental distribution (*p* value <0.0001).

In Aydin's study, the most common distribution type among the malignant lesions was segmental distribution (40%). This difference was statistically significant (*p* value = 0.001).

Similar to our study, a linear distribution was also observed mainly in benign NME (35.4%) and was statistically significant (*p* value = 0.002).

He found a significant association between malignancy and diffuse distribution (*p* value = 0.039).

However, this may be due to the low number of cases with diffuse distribution [21].

Liu et al. found that most malignant NME lesions (42.9%) depict segmental distribution (*p* = 0.01).

The linear distribution was more common in benign lesions (19.4%) than that in the malignant NME group (1/56, 1.8%), and the difference was statistically significant (*p* value = 0.002).

They also found that diffuse distribution was significantly associated with invasion (*p* value = 0.023). This may be due to the larger average diameter of the invasive lesions compared to DCIS [22].

In our study, most malignant NMEs (77.8%) and most NMEs with pathologically proven invasive cancer (92.3%) showed no local distribution, which compared with benign NMEs, and were statistically different (*p* value <0.05). This suggests that this is a more favourable distribution pattern.

Previous studies have reported a wide range of PPV results for focal and linear distributions. However, these values were lower than those of the segmental distribution [21, 22].

Regarding the internal enhancement features of NME, previous studies have reported that clustered ring enhancement can effectively recognize malignant NME lesions [21, 23].

Similarly, in our study, all NME lesions with clustered ring internal enhancement pattern proved to be malignant, whereas, none of the benign NMEs showed statistically significant clustered ring enhancement (*p* value = 0.001).

In the study by Liu et al., clustered ring enhancement was detected in 33.9% (19/56) of malignant NME lesions and 4.8% (3/62) of benign NME lesions and was statistically significant (*p* value <0.001) [22].

Similarly, Yang et al. reported a strong association between clustered ring patterns and malignancy [15].

The study by Fleury et al. also found that the most suspicious feature of NME was a clustered ring pattern and the malignant pathologic outcome predominating in the segmental distribution. The PPV value of the distribution descriptor was the highest, but not statistically significant [24].

The most common internal enhancement pattern in Aydin's study was the clumped enhancing pattern. There was no association between malignancy and clumped pattern enhancement. Homogeneous pattern was not observed in malignant lesions in their study [21]. Likewise, various studies have reported no association between malignancy and homogeneous pattern of enhancement with very low PPV values.

Similarly, in our study, we observed a homogeneous enhancement pattern in 40% of benign NMEs, whereas only 5.5% of malignant NMEs showed a homogeneous pattern, which was statistically significant (*p* value = 0.007), making it the most favourable enhancement pattern.

The most common internal enhancement of NME in our study was a heterogeneous pattern in both malignant (55.5%) and benign (46.7%) NME cases without a statistically significant difference, making it difficult to distinguish between benign and malignant lesions.

Only in Torous et al.'s study [16], no statistically significant difference was found in distribution pattern of benign or malignant NME cases, and malignant lesions were significantly more often associated with homogeneous or clumped internal enhancement in contrary to other aforementioned studies.

The reported predictive value of internal enhancement and distribution patterns differs in various studies. This may be due to the different sizes of study groups and selection bias since some studies only assessed NMEs which underwent biopsy.

Segmental distribution and clustered ring enhancement were the most important predictors of malignancy in our NME cases, which is consistent with the results of previous studies such as Lunkiewicz et al. [23], Liu et al., and Aydin's studies [21, 22].

In regard to the correlation between invasive behaviour of the malignant NME lesions and the enhancement pattern found in NME, Machida et al. found that clustered ring enhancement demonstrates a significant association with invasion [25]. Hahn et al. also reported that clustered ring pattern was more frequently observed in microinvasive ductal carcinoma than in pure DCIS [26].

In our study and the study by Liu et al., there was no difference in internal enhancement between invasive carcinoma and carcinoma in situ, probably due to the small sample size [22].

Regarding the time-intensity curve (TIC) of NME lesions, Goto et al. found that a kinetic curve was less beneficial for assessment of NME lesions than mass lesions [27]. In Gang Liu et al.'s study, malignant NME lesions mostly showed a washout type 3 curve, and benign NME lesions often depicted a persistent type 1 dynamic curve, which was statistically significant (*p* value <0.05) [22]. In Torous et al.'s study, malignant NME lesions were significantly associated with a type 3 curve (*p* value = 0.01) [16]. In the study of Yang et al. and also Aydin's study, the most frequent curve type among malignant lesions was the type 2 curve [15, 21].

In our study, all NME cases with plateau or washout TIC (type 2 and type 3) were malignant in pathology reports, and all benign NME cases had persistent enhancement (type 1) in kinetic curve with statistically significant difference (*p* value <0.0001).

We also evaluated the kind of dynamic curve types in invasive cancer and carcinoma in situ cases. None of the noninvasive NME lesions showed washout dynamic type, and some of the invasive carcinoma (38.5%) had washout dynamic curve; however, it was not statistically significant (*p* value = 0.1).

Similarly, Liu et al. found that the washout dynamic type was more frequent in invasive carcinoma (75%), which was substantially higher than in the DCIS group (31.3%) (*p* value = 0.001) which indicates that washout dynamic type of NME can potentially differentiate invasive from pure carcinoma in situ cases [22].

Although the apparent diffusion coefficient (ADC) value is useful to differentiate benign and malignant lesions in mass lesions of the breast [28, 29], some studies suggest that it is not reliable for evaluation of NME lesions [30].

In the current study, diffusion restriction on DWI sequence was present in 89% of malignant NME lesions which was statistically significant (*p* value <0.0001) compared with benign NMEs similar to Aydin's study [21].

We also found that the DWI sequences and ADC value are beneficial to differentiate malignant from benign NME lesions. Malignant NME lesions showed lower ADC values (1.28 × 10 − 3 mm^2^/s) in comparison to benign lesions (1.63 × 10 − 3 mm^2^/s) with statistically significant difference (*p* value <0.0001), which is comparable to Liu et al.'s study [22].

In some previous studies, the ADC number cut-off point (1.3 × 10 − 3 mm^2^/s) was used for differentiation between benign and malignant lesions [22, 31].

In the contrary, Avendano et al. showed that the ADC value was not valuable to discriminate between benign and malignant NME lesions [32]. In Liu et al.'s study, the degree of restricted diffusion on DWI in malignant NME lesions was higher than that in benign NME lesions, but there was no statistically significant difference (*p* value = 0.248) [22].

The differences of the results might be due to the various ROI measurement methods and therefore should be interpreted with caution.

Additionally, we noticed that invasive carcinoma demonstrates restricted diffusion on DWI more likely than noninvasive cancer, and the average of ADC values was lower in invasive cancer than that in carcinoma in situ, but their differences were not statistically significant (*p* value = 0.06, *p* value = 0.17).

Liu et al. found that the mean ADC value of invasive carcinoma (0.933 × 10 − 3 mm^2^/s) was statistically lower than that of carcinoma in situ (1.13 × 10 − 3 mm^2^/s), and restricted diffusion on DWI was statistically different between invasive cancer and DCIS (*p* value <0.05) [22].

These different results could be attributable to a diverse sample size.

This study had some limitations, including its retrospective nature and small sample size, and further studies with larger sample sizes are needed to confirm the conclusions. Another limitation of the study is that the biopsies were not performed under MRI guidance.

## 5. Conclusions

This study demonstrated that DCE-MRI is useful to predict the malignant versus benign NME lesions. Segmental distribution, clustered ring enhancement, type 2 or type 3 dynamic curve, restricted diffusion on DWI, and lower ADC value are significantly more associated with malignancy.

## Figures and Tables

**Table 1:** NME (nonmass enhancement) features classified as probably benign or suspicious.

NME features	Probably benign NME	Suspicious NME
Distribution	Focal or regional	Linear or segmental
Internal enhancement	Heterogeneous or homogenous	Clumped or clustered ring
TIC	Medium-slow early enhancement with persistent delayed curve	Fast early enhancement and delayed plateau or washout pattern
DWI/ADC	Absent restricted diffusion	Present restricted diffusion

**Table 2:** MR imaging descriptors in NME (nonmass enhancement) lesions.

Descriptor	Malignant (*n* = 18) (%)	Benign (*n* = 45) (%)	*p* value
Distribution			
Focal	4 (22.2)	24 (53.4)	0.023
Linear	0 (0.0)	11 (24.4)	0.01
Segmental	12 (66.7)	8 (17.7)	0.0001
Regional	0 (0.0)	2 (4.5)	0.08
Diffuse	2 (11.1)	0 (0.0)	0.078
Internal enhancement patterns			
Heterogeneous	10 (55.5)	21 (46.7)	0.3
Clustered ring	4 (22.3)	0 (0.0)	0.001
Clumped	3 (16.7)	6 (13.3)	0.7
Homogeneous	1 (5.5)	18 (40)	0.007
Kinetic curve			
Persistent	3 (16.7)	45 (100)	0.0001
Plateau	10 (55.5)	0 (0.0)	0.0001
Washout	5 (27.8)	0 (0.0)	0.0001
Diffusion restriction			
Present	16 (89)	2 (11.1)	0.0001
Absent	2 (11)	43 (95.6)	

## Data Availability

Any data not presented will be made available upon request.
